# Prognostic Value of Elevated Cardiac Troponin I After Aneurysmal Subarachnoid Hemorrhage

**DOI:** 10.3389/fneur.2021.677961

**Published:** 2021-05-31

**Authors:** Fa Lin, Yu Chen, Qiheng He, Chaofan Zeng, Chaoqi Zhang, Xiaolin Chen, Yuanli Zhao, Shuo Wang, Jizong Zhao

**Affiliations:** ^1^Department of Neurosurgery, Beijing Tiantan Hospital, Capital Medical University, Beijing, China; ^2^China National Clinical Research Center for Neurological Diseases, Beijing, China; ^3^Center of Stroke, Beijing Institute for Brain Disorders, Beijing, China; ^4^Beijing Key Laboratory of Translational Medicine for Cerebrovascular Disease, Beijing, China; ^5^Savaid Medical School, University of the Chinese Academy of Sciences, Beijing, China

**Keywords:** aneurysmal subarachnoid hemorrhage, troponin, prognostic, major adverse cardiac event, outcome

## Abstract

**Object:** Patients with aneurysmal subarachnoid hemorrhage (aSAH) have an increased incidence of cardiac events and short-term unfavorable neurological outcomes during the acute phase of bleeding. We studied whether troponin I elevation after ictus can predict future major adverse cardiac events (MACEs) and long-term neurological outcomes after 2 years.

**Methods:** Consecutive aSAH patients within 3 days of bleeding were eligible for review from a prospective observational cohort (ClinicalTrials.gov Identifier: NCT04785976). Potential predictors of future MACEs and unfavorable long-term neurological outcomes were calculated by Cox and logistic regression analyses. Additional Kaplan–Meier curves were performed.

**Results:** A total of 213 patients were enrolled with an average follow-up duration of 34.3 months. Individuals were divided into two groups: elevated cTnI group and unelevated cTnI group. By the last available follow-up, 20 patients had died, with an overall all-cause mortality rate of 9.4% and an annual all-cause mortality rate of 3.8%. Patients with elevated cTnI had a significantly higher risk of future MACEs (10.6 vs. 2.1%, *p* = 0.024, and 95% CI: 1.256–23.875) and unfavorable neurological outcomes at discharge, 3-month, 1-, 2-years, and last follow-up (*p* = 0.001, *p* < 0.001, *p* = 0.001, *p* < 0.001, and *p* < 0.001, respectively). In the Cox analysis for future MACE, elevated cTnI was the only independent predictor (HR = 5.980; 95% CI: 1.428–25.407, and *p* = 0.014). In the multivariable logistic analysis for unfavorable neurological outcomes, peak cTnI was significant (OR = 2.951; 95% CI: 1.376–6.323; *p* = 0.005). Kaplan–Meier analysis indicated that the elevated cTnI was correlated with future MACE (log-rank test, *p* = 0.007) and subsequent death (log-rank test, *p* = 0.004).

**Conclusion:** cTnI elevation after aSAH could predict future MACEs and unfavorable neurological outcomes.

## Introduction

The brain-heart connection loses balance after stroke (1). Cardiac complication has been shown to frequently occur in the emergency aneurysmal subarachnoid hemorrhage (aSAH) (2–4). A series of previous studies have shown that circulating cardiac biomarkers, including creatine phosphokinase isoenzyme-MB (CK-MB), troponin, brain natriuretic peptide (BNP), and N-terminal pro-B-type natriuretic peptide (NT-proBNP), are associated with delayed cerebral ischemia (DCI), short-term unfavorable neurological outcomes, and in-hospital mortality at the acute phase after aSAH (5–8). Among the cardiac biomarkers listed above, the incremental level of cardiac troponin I (cTnI) on admission was reported in 21–68% of emergency aSAH patients (6, 9), and the cardiac troponin has been shown to reach high sensitivity and specificity in the identification of cardiac abnormalities indicating subsequent major adverse cardiac events (MACEs) at the acute phase of aSAH, though conflicting results have been reported (2, 3, 10–12).

However, the long-term prognostic value of troponin elevation after emergency aSAH remains unclear. In this study, we aimed to explore whether the admission cTnI of emergency aSAH patient at the acute phase could predict future MACEs and long-term unfavorable neurological outcomes.

## Materials and Methods

### Study Design

**Figure 1:**
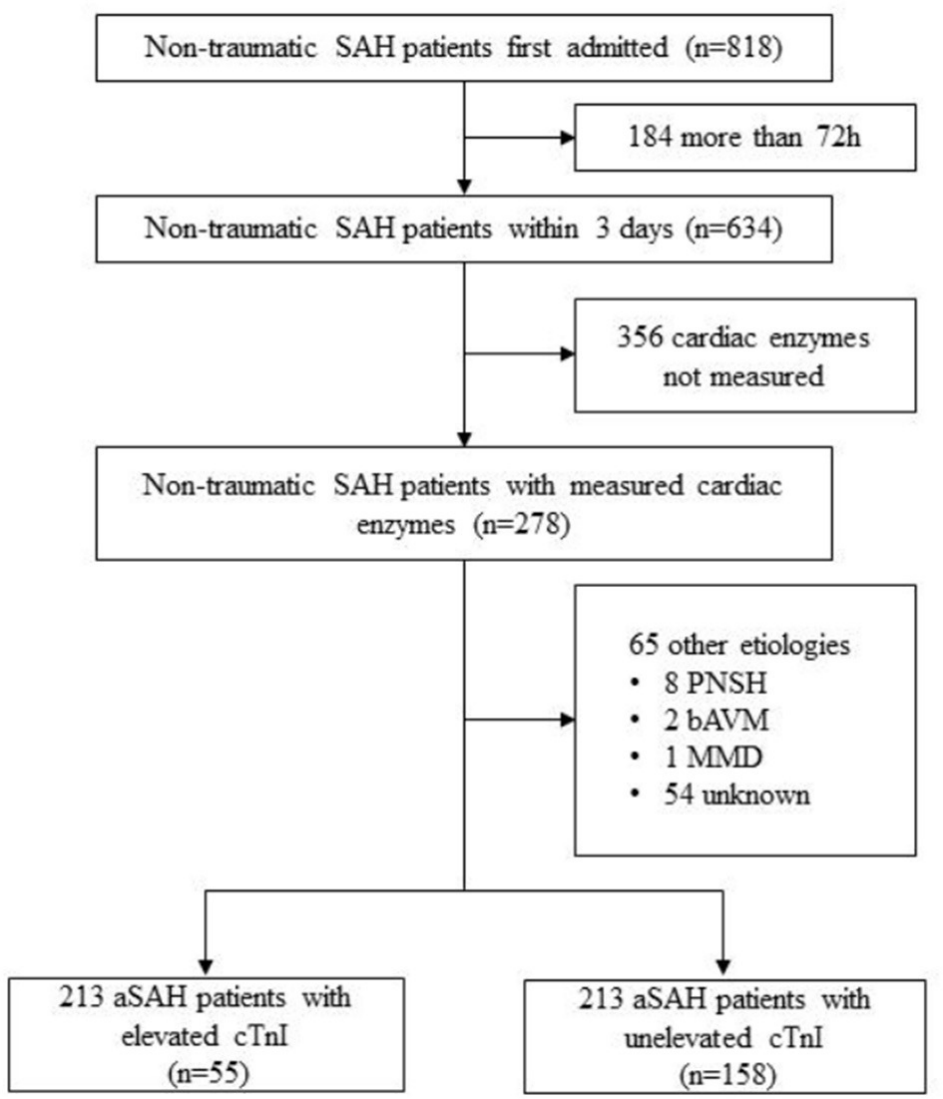
Flow chart showing inclusion/ exclusion of patients.

Consecutive aSAH patients who conducted cardiac enzymes laboratory tests within 72 h of bleeding were eligible for review from a single-center prospective cohort study of intracranial aneurysms in Beijing Tiantan Hospital between January 2016 and December 2017 (Figure 1; ClinicalTrials.gov Identifier: NCT04785976). The diagnosis confirmed at the first interview was based on the 2012 guidelines for aSAH (13).

### Data Collection and Definitions

Clinical data were retrospectively reviewed, including demographic, laboratory, radiological, and treatment-related information. Comorbid conditions were taken into consideration and conducted by the Charlson Comorbidity Index (CCI). The neurological status was evaluated by the modified Rankin Scale (mRS), and the clinical severity of SAH was assessed by the Hunt-Hess scale. Imaging characteristics, including Fisher score and intraventricular hemorrhage (IVH) involvement, were verified by at least two radiologists who had more than 5 years of clinical experience in the radiology center of our institute. The highest level of cTnI within 3 days after the rupture event was selected as the parameter of interest. Diverse cTnI assays were applied for the last decades. And according to the result of cardiac enzyme kit used in this study period (the lower limits of detectable cTnI level, 0.016 ng/mL), patients were dichotomized into two groups: the cTnI elevated group (cTnI value > 0.016 ng/mL) and the cTnI unelevated group (cTnI value ≤ 0.016 ng/mL).

In-hospital complications were defined as major adverse cardiac events (MACEs, with the occurrence of an arrhythmia, myocardial injury, acute heart failure, repeat revascularization, and cardiac arrest, or as defined in our electronic medical records) (14), novel DCI (15)/cerebral infarction (CI), hydrocephalus, seizure, intracranial infection (ICI), pneumonia, and deep venous thrombosis (DVT). The primary goal of interest was future MACEs occurring after discharge. The secondary goal was dichotomized into favorable (mRS of 0–3) and unfavorable (mRS of 4–6). Follow-up was conducted in the first 3–6 months and annually after surgery by clinical visits and telephone interviews. Evaluation of the MACEs and mRS score was conducted by neurosurgeons who had at least 5 years' experience in clinical practice, and a training program was administered to ensure the measurement accuracy. Researchers who performed follow-up assessments were blinded to the different cTnI subgroups. Patients who were lost to follow-up were not included in the prognostic analyses of 3-month, 1-, and 2-year, but not excluded at their last available follow-up from the statistical analysis.

### Statistical Analysis

The categorical variables are presented as counts (with percentages), and the continuous variables are presented as the means ± standard deviations (SD). Two-tailed *t*-tests were used for the continuous variable with Gaussian distribution. The Mann–Whitney *U* (Wilcoxon) test was used to compare non-normal distribution continuous variables. The Pearson chi-square test or Fisher exact test was used to compare categorical variables as appropriate. Multivariable Cox regression model was used to examine the risk factors associated with future MACEs. A multivariable logistic regression model was built to predict the unfavorable neurological outcomes based on the covariables, including age, sex, Fisher score (ordinal), Hunt-Hess scale on admission (ordinal), and troponin elevation (dichotomized). Hazard ratios (HRs) or odds ratios (ORs) and 95% confidence intervals (CI) for potential risk factors of MACEs and unfavorable mRS were calculated. Variables' *p* < 0.10 in univariate analysis were selected for the multivariate model using a manual forward model building strategy. Kaplan–Meier curves with log-rank were performed to estimate the risk of MACE and death after aSAH for the dichotomized troponin elevation groups. A *p* < 0.05 (two-sided) was considered as statistically significant. All statistical analyses were performed with SPSS for Windows (version 25.0; IBM, New York, USA).

## Results

A total of 213 cases of aSAH that met the inclusion criteria were included between January 2016 and December 2017. After the rupture event occurred, the highest level of troponin was analyzed (9, 16) 159 patients (74.6%) received troponin tests on post-hemorrhagic day 1, 36 (16.9%) on day 2, and 18 (8.5%) on day 3. Individuals were divided into two groups based on whether cTnI value elevated or not, namely elevated cTnI group (*n* = 55, 25.8%) and unelevated cTnI group (*n* = 158, 74.2%).

### Baseline Characteristics

The baseline characteristics of the 213 aSAH patients were summarized in Table 1. The mean (±SD) age was 57.4 ± 12.1 years, with an average follow-up duration of 34.3 months. 71.4% of them were evaluated as Hunt-Hess grade 1–2 on admission, and 57.7% were classified as Fisher score 1–2 based on the admission CT scan. Most patients (61.5%) had preoperative hypertension, and 36 (16.9%) had a confirmed cardiac disease history, including 33 (15.5%) coronary artery disease, two (0.9%) heart failure, and five (2.3%) arrhythmia. Only 14 cases (6.6%) took ACEI/ARB drugs regularly before the rupture event. The percentage of other elevated cardiac laboratory indicators was 53.1% (BNP), 25.4% (CK-MB), and 31.0% (Myoglobin, Myo), respectively. Finally, 18 cases (8.4%) received conservative management, 49.8% underwent craniotomy clipping, and 41.8% received endovascular embolization.

**Table 1:** Baseline characteristics between patients with or without elevation of troponin I.

**Variable**	**Total (%)**	**Elevated cTnI (%)**	**Unelevated cTnI (%)**	***P*-value**
	***n* = 213**	***n* = 55**	***n* = 158**	
Age (Years)	57.4 ± 12.1	59.3 ± 11.2	56.8 ± 12.4	0.192
Female	131/213 (61.5)	44/55 (80.0)	87/158 (55.1)	0.001
**Medical history**				
Diabetes mellitus	17 (8.0)	4 (7.3)	13 (8.2)	1.000
Hypertension	131 (61.5)	31 (56.4)	100 (63.3)	0.363
Dyslipidemia	27 (12.7)	10 (18.2)	17 (10.8)	0.154
Hyperhomocycteinemia	17 (8.0)	2 (3.6)	15 (9.5)	0.275
Stroke	36 (16.9)	11 (20.0)	25 (15.8)	0.476
^*^CCI-i	3.4 ± 2.0	3.7 ± 2.0	3.3 ± 2.0	0.193
**Cardiac history**				
Coronary artery disease	33 (15.5)	10 (18.2)	23 (14.6)	0.522
Heart failure	2 (0.9)	1 (1.8)	1 (0.6)	0.451
Arrhythmia	5 (2.3)	1 (1.8)	4 (2.5)	1.000
History of brain operation	1 (0.5)	1 (1.8)	0 (0)	0.258
History of cardiac surgery	9 (4.2)	1 (1.8)	8 (5.1)	0.521
ACEI/ARB	14 (6.6)	2 (3.6)	12 (7.6)	0.481
Anticoagulant drugs	2 (0.9)	1 (1.8)	1 (0.6)	0.451
Antiplatelet drugs	20 (9.4)	4 (7.3)	16 (10.1)	0.532
peak cTnI	0.007 (0.002–0.075)	0.366 (0.058–1.380)	0.002 (0.001–0.007)	0.000
Elevated BNP	113 (53.1)	46 (83.6)	67 (42.4)	0.000
Elevated CK-MB	54 (25.4)	35 (63.6)	19 (12.0)	0.000
Elevated Myo	66 (31.0)	32 (58.2)	34 (21.5)	0.000
Hunt-Hess grade				0.000
1–2	152 (71.4)	27 (49.1)	125 (79.1)	
3–5	61 (28.6)	28 (50.9)	33 (20.9)	
Fisher score				0.014
1–2	123 (57.7)	24 (43.6)	99 (62.7)	
3–4	90 (42.3)	31 (56.4)	59 (37.7)	
IVH involvement	121 (56.8)	35 (63.6)	86 (54.4)	0.235
Treatment				0.175
Microsurgery	106 (49.8)	24 (43.6)	82 (51.9)	
Endovascular treatment	89 (41.8)	23 (41.8)	66 (41.8)	
Medication	18 (8.4)	8 (14.5)	10 (6.3)	

* *CCI-i, Charlson Comorbidity Index-i; BNP, brain natriuretic peptide; CK-MB, creatine phosphokinase isoenzyme—MB; Myo, myoglobin; ACEI, angiotensin-converting enzyme inhibitors; ARB, angiotensin receptor blocker; IVH, intraventricular hemorrhage; peak.cTnI, peak value of cardiac troponin I*.

In the subgroup comparison, age, cardiac diseases history, and CCI did not have significant correlations with the elevated cTnI after aSAH (59.3 ± 11.2 vs. 56.8 ± 12.4, *p* = 0.192; 18.2 vs. 16.5, *p* = 0.769; 3.7 ± 2.0 vs. 3.3 ± 2.0, *p* = 0.193, respectively). However, patients with elevated cTnI were more likely to be female (80.0 vs. 55.1%, *p* = 0.001), have a higher Hunt-Hess grade (HH grade 3–5, 50.9 vs. 20.9%, and *p* < 0.001), and higher Fisher score (Fisher score 3–4, 56.4 vs. 37.7%, and *p* = 0.014). Other cardiac laboratory indicators were synchronized with the elevated cTnI (BNP, 83.6 vs. 42.4%, *p* < 0.001; CK-MB, 63.6 vs. 12.0%, *p* < 0.001; Myo, 58.2 vs. 21.5%, *p* < 0.001; respectively). There were no significantly statistical differences in other demographic characteristics and radiological matters in patients with and without detectable elevation of cTnI.

### Outcomes

Clinical outcomes were presented in Table 2. The common in-hospital complications were pneumonia (72 patients, 33.8%) and DVT (65 patients, 30.5%). Of 61 (28.6%) patients who developed MACEs during hospitalization, 30 patients (14.1%) had acute heart failures, 27 (12.7%) had arrythmia, 19 (8.9%) had myocardial injuries, and two patients (0.9%) experienced cardiac arrests. In addition, we found a higher incidence of MACE (43.6 vs. 23.4%, *p* = 0.004), DCI (30.9 vs. 13.9%, *p* = 0.005), and DVT (49.1 vs. 24.1%, *p* = 0.001) in the elevated cTnI group. The mortality during hospitalization in the whole cohort was 3.8% (*n* = 8; elevated cTnI group vs. unelevated cTnI group: 10.9 vs. 1.3%, *p* = 0.001).

**Table 2:** Outcomes between patients with or without elevation of troponin I.

**Variable**	**Total (%)**	**Elevated cTnI (%)**	**Unelevated cTnI (%)**	***P*-value**
	***n* = 213**	***n* = 55**	***n* = 158**	
**In-hospital complications**				
^*^MACE	61 (28.6)	24 (43.6)	37 (23.4)	0.004
Myocardial injury	19 (8.9)	10 (18.2)	9 (5.7)	0.012
Acute heart failure	30 (14.1)	15 (27.3)	15 (9.5)	0.001
Arrythmia	27 (12.7)	5 (9.1)	22 (13.9)	0.353
Cardiac arrest	2 (0.9)	1 (1.8)	1 (0.6)	1.000
DCI/CI	39 (18.3)	17 (30.9)	22 (13.9)	0.005
Hydrocephalus	15 (7.0)	6 (10.9)	9 (5.7)	0.320
Seizure	6 (2.8)	2 (3.6)	4 (2.5)	1.000
ICI	16 (7.5)	3 (5.5)	13 (8.2)	0.708
Pneumonia	72 (33.8)	20 (36.4)	52 (32.9)	0.631
DVT	65 (30.5)	27 (49.1)	38 (24.1)	0.001
Hospitalization duration	15.5 ± 10.2	16.7 ± 10.0	15.1 ± 10.3	0.338
In-hospital mortality	8 (3.8)	6 (10.9)	2 (1.3)	0.001
Discharge mRS > 3	60/213 (28.2)	25/55 (45.5)	35/158 (22.2)	0.001
3-month mRS > 3	37/187 (19.8)	18/46 (39.1)	19/141 (13.5)	0.000
1-year mRS > 3	38/187 (20.3)	17/46 (37.0)	21/141 (14.9)	0.001
2-year mRS > 3	35/186 (18.8)	18/46 (39.1)	17/140 (12.1)	0.000
Last follow-up mRS > 3	46/213 (21.6)	23/55 (41.8)	23/158 (14.6)	0.000

* *MACE, major adverse cardiac event; DCI, delayed cerebral ischemia; CI, cerebral infarction; ICI, intracranial infection; DVT, deep venous thrombosis*.

**Figure 2:**
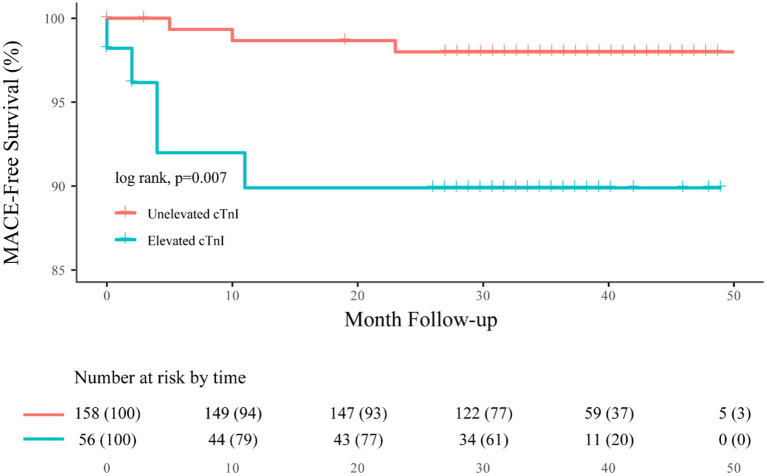
Overall Kaplan–Meier estimates for freedom from MACE, comparing patients who had elevated cTnI (blue line) with unelevated cTnI (orange line; log-rank test, *p* = 0.007).

After discharge, eight future MACEs occurred in seven patients (3.3%), yielding an annual incidence of cardiac events of 1.5%. Patients with elevated cTnI had a significantly higher rate of future MACEs than the unelevated group (10.6 vs. 2.1%, *p* = 0.024, and 95% CI: 1.256–23.875). This finding was also statistically significant in the Kaplan–Meier analysis between these two groups (log-rank, *p* = 0.007; Figure 2). In addition, we found that MACEs mostly occurred within 1 year after aneurysmal rupture (seven in the first year, one in the second year).

**Figure 3:**
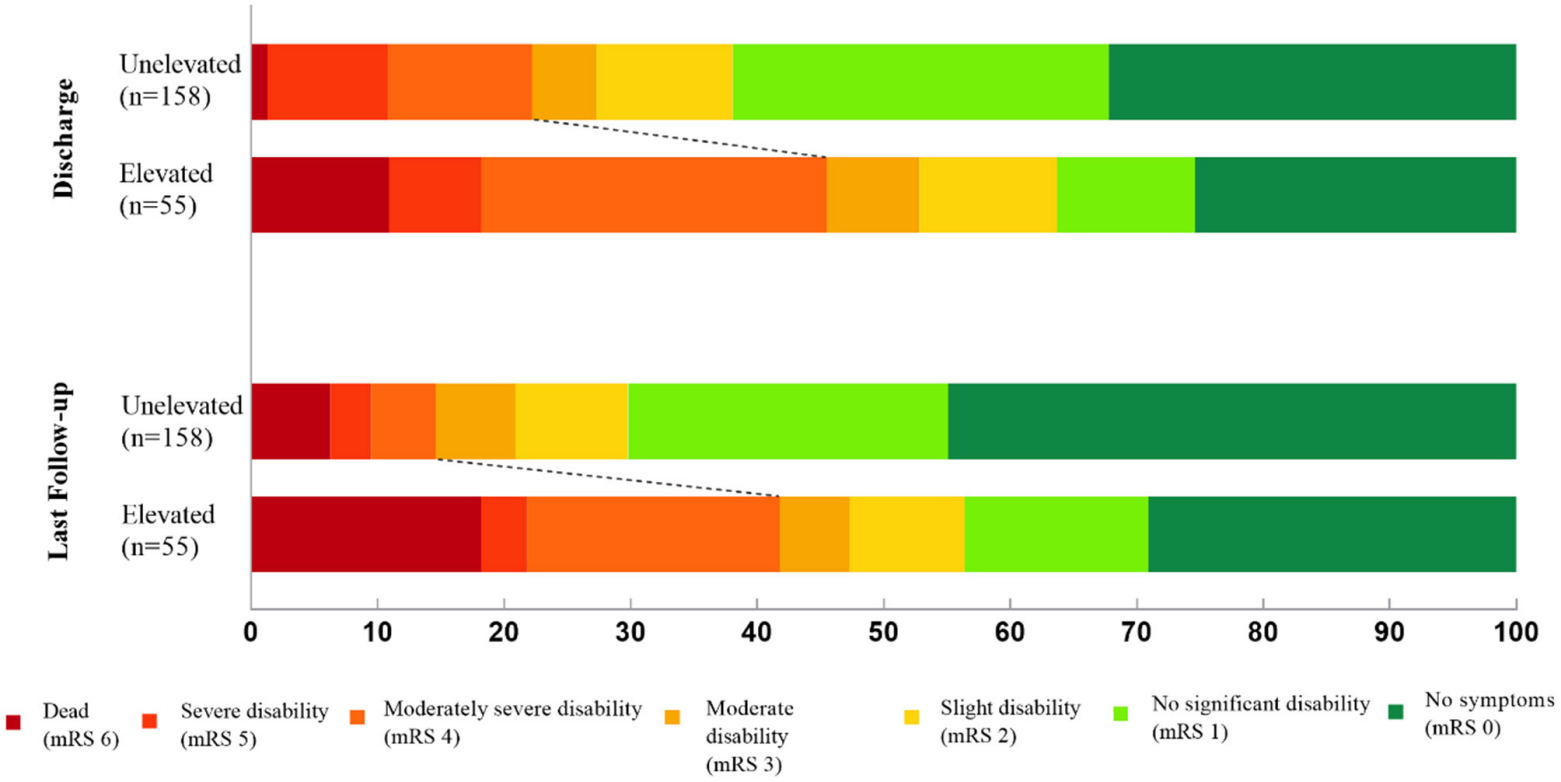
Distribution of modified Rankin Scale (mRS) scores at discharge and last follow-up after aneurysmal subarachnoid hemorrhage.

**Figure 4:**
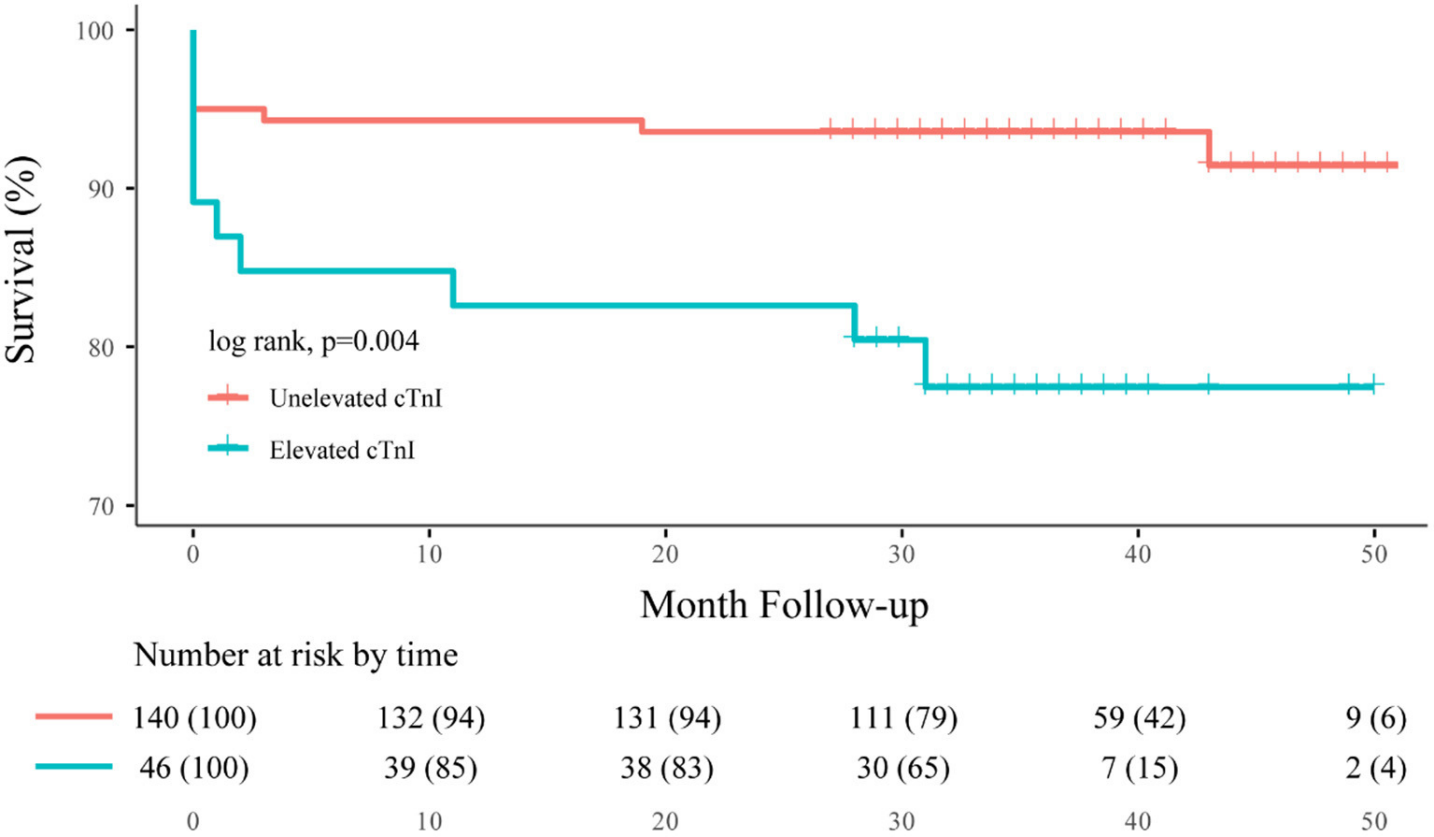
Overall Kaplan–Meier estimates for freedom from death, comparing patients who had elevated cTnI (blue line) vs. unelevated cTnI (orange line; log-rank test, *p* = 0.004).

During the clinical follow-up, we observed a significant correlation between elevated cTnI and unfavorable neurological outcomes (mRS > 3) at discharge, 3-month, 1-, 2-year, and last follow-up (45.5 vs. 22.2%, *p* = 0.001; 39.1 vs. 13.5%, *p* < 0.001; 37.0 vs. 14.9%, *p* = 0.001; 39.1 vs. 12.8%, *p* < 0.001; 41.8 vs. 14.6%, *p* < 0.001, respectively; Figure 3). By the last available follow-up, 20 patients had died, with an overall mortality of 9.4% and an annual death rate of 3.8% (8.9 vs. 2.3%, *p* = 0.005). In addition, the Kaplan–Meier analysis of mortality during the clinical follow-up was 21.7% with elevated cTnI and 7.1% without (log-rank, *p* = 0.004; Figure 4).

### Predictors of Future MACEs and Unfavorable Long-Term Outcomes

A total of 27 patients were lost to follow-up, namely 26 after discharge and one at 1-year, with a missing rate of 12.7%. The remaining 186 patients were included in the survival analysis, amounting to 532 follow-up patient-years.

During hospitalization, 61 (28.6%) patients experienced MACEs. The univariable Cox analysis showed that age (HR = 0.927; 95% CI: 0.861–0.998, and *p* = 0.044), elevated cTnI (HR = 9.603; 95% CI: 1.487–55.209, and *p* = 0.016), and in-hospital pneumonia (HR = 0.104; 95% CI: 0.013–0.837, and *p* = 0.033) were significantly correlated with future MACEs (Table 3). When clinical confounding variables (age by decades, Hunt-Hess scale, Fisher score, and in-hospitalization complications) were incorporated into the multivariable Cox regression model, only elevated cTnI was statistically significant (HR = 5.974; 95% CI: 1.426–25.019, and *p* = 0.014).

**Table 3:** Cox regression analysis of predictors for future MACEs.

	**Primary outcome characteristics**		**Univariate**		**Multivariate**	
**Variable**	**MACE**	**Non-MACE**	***p*-value**	**HR (95% CI)**	***p*-value**	**OR (95% CI)**
Age, years	53.5 ± 9.8	58.0 ± 12.5	0.044	0.927 (0.861–0.998)	0.238	
Gender	6 (75.0)	113 (62.8)	0.184	6.613 (0.405–107.921)		
HH grade 3–5	3 (37.5)	51 (28.3)	0.248	0.222 (0.017–2.862)		
^*^FS 3–4	4 (50.0)	73 (40.6)	0.728	1.436 (0.185–11.13)		
IVH involvement	5 (62.5)	101 (56.1)	0.518	1.818 (0.295–11.191)		
Smoke	2 (25.0)	56 (31.1)	0.43	0.388 (0.037–4.079)		
History of stroke	2 (25.0)	31 (17.2)	0.226	0.302 (0.043–2.098)		
Hypertension	4 (50.0)	112 (62.2)	0.239	3.31 (0.449–24.361)		
HR	82.9 ± 18.1	75.6 ± 13.5	0.405	1.027 (0.964–1.093)		
SBP	159.9 ± 26.0	161.3 ± 24.2	0.634	1.008 (0.975–1.042)		
Elevated cTnI	5 (62.5)	42 (23.3)	0.016	9.063 (1.487–55.209)	0.014	5.980 (1.428–25.407)
ih.MACE	5 (62.5)	49 (27.2)	0.064	4.528 (0.91–22.512)	0.064	
ih.DCI/CI	1 (12.5)	33 (18.3)	0.246	0.236 (0.02–2.711)		
ih.pneumonia	4 (50.0)	58 (32.2)	0.033	0.104 (0.013–0.837)	0.211	
ih.DVT	4 (50.0)	52 (28.9)	0.512	0.521 (0.074–3.656)	0.622	

* *FS, Fisher score; HR, admission heart rate; SBP, admission systolic blood pressure; cTnI, cardiac troponin I; ih.MACE, in hospitalization major adverse cardiac event; ih.DCI/CI, in hospitalization delayed cerebral ischemia/ cerebral infarction; ih.pneumonia, in hospitalization pneumonia; ih.DVT, in hospitalization deep venous thrombosis*.

The univariable logistic analysis showed that elevated cTnI was associated with unfavorable long-term outcomes at the last follow-up (OR = 4.218; 95% CI: 2.106–8.450; *p* < 0.001). Age, history of stroke, Hunt-Hess grade 3–5, Fisher score 3–4, perioperative pneumonia, and DVT had a *p* < 0.10. Those variables were verified to have had a major impact on outcome in previous studies (13) and were selected in the reduced regression model. In the multivariable analysis, peak cTnI (OR = 2.951; 95% CI: 1.376–6.323; *p* = 0.005), age (OR = 1.046; 95% CI: 1.012–1.081; *p* = 0.008), and Hunt-Hess grade (OR = 4.017; 95% CI: 1.909–8.453; *p* < 0.001) were independent predictors for unfavorable long-term outcomes (Table 4).

**Table 4:** Logistic regression analysis of predictors for last follow-up outcomes.

	**Last follow-up outcomes**		**Univariate**		**Multivariate**	
**Variable**	**Unfavorable**	**Favorable**	***p*-value**	**OR (95% CI)**	***p*-value**	**OR (95% CI)**
Age, years	62.5 ± 12.1	56.0 ± 11.8	0.002	1.049 (1.018–1.081)	0.008	1.046 (1.012–1.081)
Gender	31 (67.4)	100 (59.9)	0.355	1.384 (0.694–2.759)		
HH grade 3–5	27 (58.7)	34 (20.4)	0.000	5.558 (2.767–11.160)	0.000	4.017 (1.909–8.453)
^*^FS 3–4	30 (65.2)	60 (35.9)	0.001	3.343 (1.686–6.627)		
IVH involvement	28 (60.9)	93 (55.7)	0.530	0.807 (0.414–1.572)		
Smoke	12 (26.1)	59 (35.3)	0.241	1.547 (0.745–3.213)		
History of stroke	12 (26.1)	24 (14.4)	0.064	0.475 (0.216–1.045)		
Hypertension	32 (69.6)	99 (59.3)	0.206	0.636 (0.316–1.282)		
HR	76.9 ± 14.5	74.8 ± 13.2	0.353	1.011 (0.987–1.036)		
SBP	162.1 ± 26.3	161.2 ± 24.3	0.813	1.001 (0.988–1.014)		
Elevated cTnI	23 (50.0)	32 (19.2)	0.000	4.218 (2.106–8.450)	0.005	2.951 (1.377–6.323)
ih.MACE	15 (32.6)	46 (27.5)	0.502	1.272 (0.629–2.572)		
ih.DCI/CI	10 (21.7)	29 (17.4)	0.498	1.321 (0.589–2.962)		
ih.pneumonia	25 (54.3)	47 (28.1)	0.001	0.329 (0.168–0.643)		
ih.DVT	21 (45.7)	44 (26.3)	0.013	0.425 (0.216–0.836)		

* *FS, Fisher score; HR, admission heart rate; SBP, admission systolic blood pressure; cTnI, cardiac troponin I; ih.MACE, in hospitalization major adverse cardiac event; ih.DCI/CI, in hospitalization delayed cerebral ischemia/cerebral infarction; ih.pneumonia, in hospitalization pneumonia; ih.DVT, in hospitalization deep venous thrombosis*.

## Discussion

In this study cohort involving aSAH patients with elevated cTnI on admission, brain-heart interactions were investigated. The major findings were that the predictor analysis showed that abnormal troponin level was associated with future MACEs and unfavorable long-term outcomes. Additionally, the survival analysis showed that aberrant cTnI was related to an increased risk of future MACEs and deaths after aSAH. The risk increased by 195% for death and by 498% for MACE, respectively.

The results of this study offer valuable information to emergency and intensive care clinicians to aid them in deciding whether or not future unexpected cardiac events could happen in patients with elevated cTnI during hospitalization or at follow-up (17, 18). To our knowledge, this is the most extensive series reporting survival and outcome in this particular subgroup. Meanwhile, we explained the continuous brain-heart interaction with detailed clinical data. Admittedly, subarachnoid hemorrhage-induced mortality in the acute phase was dreadful and following sublethal complications cannot be underestimated. In addition to the factors, such as DCI and DVT, which have been broadly verified by investigators, cardiac events secondary to the protopathy disturbed the diagnosis and treatment, in terms of both doctors and patients (19).

Recently, some clinicians have begun to pay attention to the impact of cardiovascular risk caused by aSAH. The cTnI elevation observed in this population is in keeping with the data produced by van der Bilt et al. (6) and Zhang et al. (7). Numerous studies have reported the reasonable predictive potential of admission elevated-troponin in aSAH patients, but lots of studies focused on the short-term neurological outcomes (7, 9, 20, 21), although several teams started to foresee the sensitivity and specificity of cTnI on long-term outcomes (7, 10, 16). However, none of the studies were performed for longer than 1 year. Studies that reported outcome separately for elevated cTnI and unelevated cTnI patients is in line with our findings at 3-month and 1-year. However, the disparity between studies is unescapable if outcome appraisal tools were applied differently (16). Furthermore, we conducted regular surveillance on surviving aSAH patients (mean follow-up of 34.3 ± 12.4 months) and found more unfavorable outcomes occurred in the elevated cTnI group (41.8 vs. 14.6%, *p* < 0.001).

Few studies have offered an insight into the predictive value of admission troponin elevation in future MACEs, which has been studied in general noncardiac surgery and ischemic stroke population by degrees (5, 22). Akkermans et al. (11) concluded that patients with postinterventional cTnI elevation have a higher risk of MACE within the first year, but failed to build a multivariable regression model, which is consistent with our findings concerning admission cTnI elevation (HR = 5.980; 95% CI: 1.428–25.407; *p* = 0.014). Furthermore, none of the above-cited studies used survival analysis to evaluate the level of admission cTnI for predicting future MACEs and unfavorable 2-year outcomes. One could speculate that our notion of relations between elevated cTnI and the primary and secondary outcome is accidental and unintentional. However, the independent prognostic value of elevated cTnI was also successfully established when adjusted for known predictors. Unexpectedly, the history of stroke and heart diseases had nothing to do with future MACEs (25.0 vs. 17.2%, *p* = 0.928; 12.5 vs. 17.8%, *p* = 1.000, respectively). We proposed that the ischemic preconditioning, a powerful endogenous mechanism, could be a rational mechanism to monitor the ischemic events in both subsequent brain and heart events (18, 23, 24).

The severity of brain injury was widely regarded as the leading cause of a poor outcome in aSAH patients. Cardiac complications are not the most important factor for the eventual outcome (20), but they are the second most important (1). Proposed mechanisms of brain-heart interaction after aSAH have grown ever more important. A more generally accepted hypothesis is that an increased sympathetic tone determines catecholamines discharge and subsequently SIC ensues (25). As a matter of fact, there are some other possible explanations regarding neurocardiac injuries, including right insular cortex damage, decreased focal and global cerebral perfusion, instable autoregulation, existed cardiac diseases, and impaired blood-brain barrier (26–28). Interestingly, the above-mentioned mechanisms could interpret our data to some extent in the early phase after aSAH, but some may question the phenomenon that troponin elevation was strongly associated with future MACEs and long-term neurological outcomes. Exogenous administration of norepinephrine (29), perioperative cardiac injury after aneurysm occlusion (11), and neurogenic stunned myocardium (30) could be possible answers. Future studies should offer further proof of whether there is a causal relationship between the onset of troponin discharge and MACEs and outcomes within 2 years.

In the case of aSAH-related cardiac dysfunction, it is acceptable to treat the underlying neurological condition. Due to the low recognition rate, misdiagnosis (19), and lack of randomized trials, additional management of neurocardiogenic injury remains entirely empirical in each individual case. Recognized ominous clinical factors including higher age (31) and unfavorable admission neurological status concerned with poor outcome were also statistically significant in the present study (higher age, OR = 1.046, 95% CI: 1.012–1.081, and *p* = 0.008; higher Hunt-Hess grade, OR = 4.017, 95% CI: 1.909–8.453, *p* < 0.001). Nevertheless, no cohort study has ever been published relating the troponin elevation to future MACEs and 2-year outcomes. The findings of this study may not only improve clinical practice but also direct secondary prevention cost-effectively.

The present study had some limitations. First, the study population was too small to fulfill an accurate prediction model, though our retrospectively reviewed cohort showed the independent predictive value on future MACEs and 2-year outcomes. Second, although previous studies suggest that intervention methods have no significant effect on troponin release, with the significant progress of endovascular and microsurgical procedures, the impact of different treatments on MACEs after aSAH needs to be further explored (22). Finally, the laboratory updated cTnI settings once during the study period, and absolute value to stratification was impractical. Emergency and intensive care unit clinicians made decisions based on the thresholds laboratory offered, so different assays did not influence our results. Further quantitative analysis of sensitivity and specificity of troponin needs to be investigated.

## Conclusion

CTnI elevation after the ictus of a ruptured intracranial aneurysm can predict the occurrence of MACEs and unfavorable outcomes within 2 years after aSAH. Although admission troponin elevation can be recognized as a biomarker to identify aSAH patients at high risk of neurocardiac injuries, further investigation into clinical management is needed to prevent cardiac complications and improve outcomes.

## Data Availability Statement

The raw data supporting the conclusions of this article will be made available by the authors, without undue reservation.

## Ethics Statement

The studies involving human participants were reviewed and approved by the Institutional Review Board of Beijing Tiantan Hospital. The patients/participants provided their written informed consent to participate in this study.

## Author Contributions

FL and YC: conception and design and drafted the article. FL, YC, QH, CZe, and CZh: acquisition of the data. CZe and CZh: analysis and interpretation of the data. XC and SW: critically revised the article. XC, YZ, SW, and JZ: reviewed the submitted version of the manuscript. All authors approved the final version of the manuscript.

## Conflict of Interest

The authors declare that the research was conducted in the absence of any commercial or financial relationships that could be construed as a potential conflict of interest.
